# The New York Institute of Stomatology

**Published:** 1904-09

**Authors:** 

**Affiliations:** The New York Institute of Stomatology


### Reports of Society Meetings.

### THE NEW YORK INSTITUTE OF STOMATOLOGY.

A REGULAR meeting of the Institute was held on Tuesday evening, May 3, 1904, at the "Chelsea," No. 222 West Twenty-third Street, New York, the President, Dr. A. H. Brockway, in the chair.

The minutes of the last meeting were read and approved.

Dr. E. A. Bogue presented the model of a combined bridge and retaining appliance for lower and upper teeth loosened by pyorrhœa. The patient had been requested to call by Dr. Barnes, of Cleveland, one of our fellow-members, the work having been done by Dr. Phillips, of Los Angeles, Cal. It consisted briefly of a lingual and buccal plate accurately fitting the teeth, screwed together, with cement between them and filling all interstices. It took sixteen days to make the upper, and nine days to make the lower, apparatus. The pyorrhœa had stopped as soon as the fixture was in place and there had been no recurrence so far as the patient could perceive.

*Dr. F. Milton Smith.*—The case presented by Dr. Bogue reminds me of an article written by Dr. William I. Fish, of Newark, nearly five years ago, and published in the INTERNATIONAL DENTAL JOURNAL, upon this subject of bracing loose teeth. That article prompted me to make a brace after this plan for the lower incisors and canines, which proved very useful; I think it would have proved more so had the patient come to me regularly to have the tartar removed. As it was, he came in after three years with the piers very loose.

*Dr. H. S. Sutphen.*—Five years ago I inserted a similar apparatus for the lower, and have seen that patient every six months since. The whole thing is somewhat loose now, because the cuspids are somewhat loose.

*Dr. L. C. LeRoy.*—A patient came into my hands for whom Dr. Fish had made a similar appliance to brace all of the upper and lower incisors and cuspids. There was also a condition of cleft palate, and consequently these upper teeth were necessary to hold the plate which was worn for its correction. The appliance strengthened the teeth to such an extent that to-day they are still doing good service after about five years. The lower ones are also doing well. I think Dr. Fish should receive all credit for giving us this excellent method.

*Dr. H. W. Gillett.*—Dr. Fossume has described a process which is often valuable in these cases. The bar is attached to incisors by dowels, the lingual portions of the cutting edges being removed to permit of so applying it that it shall be inconspicuous. This device interferes less with the process of cleaning the teeth than any other I have seen mentioned.

Dr. Freeman Allen, of Boston, read a paper entitled "Recent Methods in the Administration of Anæsthetics." Dr. Allen illustrated his paper with the various apparatus used in the different methods.

(For Dr. Allen's paper, see page 665.)

DISCUSSION.

*Dr. Emil A. Rundquist.*—The safety of anæsthesia depends upon a great many things, not the least important of which is the skill and resource of the anæsthetist. The first factor to be considered is the choice of the anæsthetic with regard to the individual and the nature of the operation. The rapidity of the operation, whether long or short, is also a factor upon which the safety of the anæsthetic depends. The preparation of the patient before the operation and the position of the patient during operation are also to be considered, a sitting position always being avoided except when administering nitrous oxide.

The choice of the anæsthetic is influenced by the climate.

Chloroform seems to be safer in hot climates, probably because it is more easily eliminated, most deaths from chloroform occurring in the cold season of the year.

Age also influences the choice. Generally speaking, children take ether more satisfactorily, while in old people chloroform is preferable. The normal adult should be given ether instead of chloroform. The pathological adult should be handled with more caution. If the blood-pressure is low, ether is indicated. In diseases of the arteries it should be borne in mind that chloroform diminishes the strain on the blood-vessels. Hence in an old person with brittle vessels chloroform is often the safer. In cases of fatty heart and myocarditis ether is indicated, but it should be administered with care. With regard to the *lungs,* chloroform is indicated in cases of chronic bronchitis or chronic tubercular lesions. In pneumonia, with poisoned heart, or empyema, ether should be given the preference. For the removal of foreign bodies in the larynx or trachea, or for the removal of tumors in these localities, chloroform is the better. Anæsthetics should be given with great care in cases of profound anæmia and blood-changes of that type. In general diseases, diabetic patients require ether. In alcoholism chloroform is better, chiefly because a person addicted to the excessive use of alcohol will require an enormous amount of ether to produce anæsthesia. In regard to the kidneys, in chronic nephritis, tension is the key note. When there is increased arterial tension, chloroform had better be given, otherwise ether is the safer, as it produces less marked degeneration. However, there is great difference of opinion on this subject. In sepsis, shock, and hemorrhage, the minimum amount of anæsthetic should be used. Ether is probably best, but must be given with care. In fat people with short necks, who generally have mechanical inspiratory difficulty, chloroform is the anæsthetic of choice.

The kind of operation influences the choice of the anæsthetic to be given. For abdominal operations Kelly prefers chloroform, but in New York the majority of men use ether. It is probable that ether is safer and gives just about as good results. In operations about the face of considerable duration, besides the methods spoken of by Dr. Allen, there is the method of administering the anæsthetic by means of a tracheotomy tube; the method of rectal anæsthesia is mentioned only to be condemned. For peripheral operations that do not require marked relaxation, in well-selected cases, laughing-gas and oxygen will do. Ethyl chloride is also used, but is not so safe, as it lowers arterial tension. In obstetrics, chloroform is generally used to ease the labor-pains, but in cases of version or Cæsarean section, ether is better. Chloroform should be avoided in cases where repeated narcoses are necessary, as chloroform is not eliminated as readily as ether, and may cause fatty changes in the viscera.

The dangers of an anæsthetic are remote and immediate. With ether there is danger of sudden respiratory paralysis causing death (rare). The remoter dangers have not been quite so clearly laid before the medical profession, but they are being met with all the time. Remote pulmonary dangers are more marked with ether than chloroform. Renal dangers may occur all the way from acute congestion with little albumin, up to suppression of urine. This occurs less with chloroform than with ether. Fatty visceral degeneration does not occur with ether. Both ether and chloroform have post-operative sequellæ, ether not causing the degeneration that is caused by chloroform. In a typical case of late chloroform death, the patient was negative to examination, but required a great deal of chloroform to produce relaxation. Convalescence went well till the second day after the operation, then jaundice appeared, with albumin and casts in the urine. There was no temperature. The patient died in coma on the fifth day. Autopsy showed fatty degeneration of all the viscera.

Of deaths from chloroform and ether, the best statistics show: ether, immediate, one in twenty-nine thousand cases; chloroform, immediate, one in three thousand cases. Remoter pulmonary deaths from ether, according to statistics from St. Thomas Hospital, London, show one in two thousand four hundred.

The causes of post-operative lung complications are: (1) Direct bronchial irritation with hypersecretion of mucous, washing down the bacteria into the terminal alveoli, setting up a pneumonia. This is worse with ether. Cyanosis distinctly increases the secretion of mucus in the bronchi. (2) Toxic action on the pulmonary blood-vessels. This is a little worse with ether than with chloroform. There is dilatation and exudation of serum. The resistance to infection by direct inspiration, emboli, and through the blood is lowered. (3) The inspiration of vomitus, blood, or infected secretions from the mouth, nose, etc. (4) Infected thrombi from operative wound. In cocaine anæsthesia in German clinics this explained the very frequently resulting pneumonia. The small thrombi were washed over into the mesenteric vessels, thence passing into the pulmonary circulation. (5) Chill, fright, or shock of operation all lower the resistance. (6) Prolonged anæsthesia even with small amounts of anæsthetic. (7) Restricted respiration from pain or tight dressings. (8) Maintenance of one position, especially in old people, may cause hypostatic pneumonia. (9) Operation or infection near pleura. The results of these post-operative lung complications may be enumerated as follows: Pulmonary œdema, acute bronchitis, lobar and broncho-pneumonia, pleurisy, hemorrhagic infarctions, gangrene, abscess and exacerbations of chronic processes, emphysema, and tuberculosis.

Several precautions are used to prevent pulmonary complications: (1) Morphine and atropine are given before an operation with ether. (2) Digitalis and strophanthus before chloroform. These are more for the effect upon the heart. (3) The stomach is washed out, and care is taken that there is not a regurgitation of the contents of the stomach, the patient virtually drowning in the vomited material. (4) Rose's position is used. (5) Interrupted ether narcosis. Sometimes possible in breast cases where complete relaxation and deep narcosis is not necessary; giving one-sixth to one-fourth grain of morphine one hour before operation and combining with local anæsthesia if necessary. (6) Becker in Hildesheimer's clinic adds twenty drops of oil of pini pumilionis to ether before beginning. In five hundred cases it stopped secretion of mucus. It has been used in cases of pulmonary disease. (7) Asepsis both of the wound and of the apparatus used. (8) The minimum amount of anæsthetic should be used. (9) The rapidity of the operation diminishes the likelihood of lung trouble. (10) Avoid undue exposure, fright, etc. (11) The patient's position in bed should be changed as frequently as possible, especially in aged patients.

Regarding the amount of anæsthetic used, out of one hundred personal cases using nitrous oxide and ether the average time of the operation was seventy-five minutes, requiring an average of 4.7 ounces of ether. This would average three and three-fourths ounces of ether per hour. Out of this number of cases the best required five ounces of ether for an anæsthesia of two hours and forty minutes. Regarding the time required for complete anæsthetization with ether used in the ordinary method, thirteen minutes is about the average, while with nitrous oxide and ether complete relaxation can be produced in an average of three minutes, and in some favorable cases in one and one-half minutes.

There is no apparatus that will make chloroform safe. Chloroform is distinctly a dangerous drug, and for the unskilled man ether is much better. I wish to deprecate the habit etherizers have of rubbing the conjunctiva to ascertain the degree of anæsthesia. It is distinctly a bad custom, because of the resulting infection and subsequent conjunctivitis. Care should be taken during operations to avoid pressures which will result often in paralysis of the musculo-spiral, brachial, or popliteal nerves. In pelvic work the anæsthesia can be pushed to stop abdominal breathing. Anæsthesia deepens somewhat in the Trendelenburg posture.

The essayist spoke of anæsthol as a very satisfactory anæsthetic. It is claimed to be a new solution, and not merely a mixture. It consists of ethyl chloride, seventeen per cent.; chloroform, thirty-six per cent.; and ether, forty per cent. It boils at 104° F. It is given on a mask by the drop method. Of course, the ideal anæsthetic would be one a certain number of minims of which, injected by a hypodermic syringe, would give a certain definite result. Some work has been done along this line. Scopolamine and morphine have been used. The two act in opposition except in the brain, where they unite to narcotize. Scopolamine is not toxic like atropine. It dilates the pupils, stimulates the pulse and respiration, stimulates peristalsis, and raises the blood-pressure. Morphine is the more dangerous drug of the two. One death has been reported in one hundred and sixty-two cases. A test dose is given the evening before of scopolamine, $^1/_{130}$ grain; morphine, ½ grain. In the morning scopolamine, $^1/_{65}$ grain, and morphine, one grain. After waiting one and one-half hours, give the evening dose again. This is the maximum dosage. If the pupils are not dilated, give more scopolamine. Blos reports one hundred and five cases, one death, seventy ideal, twenty-nine required little ether, and six could not be narcotized. The danger lies in the respiratory paralysis. The muscular relaxation is not perfect.

*Dr. Bryan D. Sheedy.*—I feel very grateful to the reader of the valuable paper presented this evening for your consideration. I also thank Dr. Wheeler, your secretary, for affording me the pleasure of hearing the paper, and your chairman for allowing me to take part in the discussion. The subject matter attracted me, though I feel very much like an outsider in taking part in this meeting, made up, as it is, almost wholly of gentlemen of another profession, and in a discussion of subjects that the medical practitioner seldom considers. As my work is limited to diseases of the mouth, ear, nose, and throat, almost daily do I come in contact with the work of the dentist. It is impossible to divorce your work from that of the rhinologists. I hoped for something in the paper and discussion that would throw light on the subject of anæsthesia of the parts which you are so often called upon to operate. The paper has certainly been a valuable and practical one along the line of general anæsthesia, and I fear the remarks I expect to make will be a little off from the point, as I intend to discuss very briefly anæsthesia as applied to the locality of our daily occupation.

One word in regard to ether and chloroform anæsthesia. In the New York Post-Graduate Medical School and Hospital, where there are probably as many surgical operations as in any other institution in New York, gas and ether anæsthesia is the rule. In London ether is seldom used. It will be a difficult matter for us to explain why most English and continental surgeons consider chloroform a safer remedy than ether, while in America ether is used almost to the complete exclusion of chloroform.

Personally I have had some very disagreeable experiences with both ether and chloroform in operations in and about the mouth, nose, and throat, and I firmly believe that if statistics were published of the operations in and about these parts under profound ether or chloroform anæsthesia, the percentage of death would be very much higher than that ordinarily given in standard text-books. I know of more than one death that has occurred in New York City during the past few years in connection with operations about the upper respiratory tract that have not been figured in estimating the percentage of deaths from anæsthetics. My personal experience in one case not very long ago will remain with me for some time. I have been teaching the students of my clinic in the New York Post-Graduate Medical School and Hospital that for short operations in and about the upper respiratory tract profound and complete anæsthesia by ether or chloroform is not advisable. I know of the arguments advanced in regard to the dangers of primary chloroform anæsthesia, but these arguments do not hold in regard to primary ether anæsthesia, as I believe the danger from complete anæsthesia in operations about the nose, throat, and mouth, and of infected secretions passing into the trachea and lungs of the patient, are not sufficiently emphasized. I recommend that just enough anæsthetic be used to prevent fright and pain, but not enough to destroy the laryngeal reflex, thereby allowing blood or infected material to gain entrance into the larynx.

I believe, if we bear this point in mind, we will not have the dangerous symptoms manifested in profound anæsthesia where bloody operations are performed in the mouth and upper respiratory tract. We will also save our patients many cases of pneumonia and other infectious diseases of the lungs following the use of general anæsthetics.

Regarding the question of chloroform and ether, I believe the average American surgeon knows very little about chloroform anæsthesia, as it is so seldom used in this country. In New York and other American cities a death from chloroform is looked upon almost as malpractice, while abroad you are immediately picked out as an American if you mention ether. The remote results of ether are far worse than we generally consider, while the immediate effect of chloroform is much better in the hands of experienced surgeons than Americans generally allow.

When we become more familiar with chloroform as it is used in Great Britain, I firmly believe we will use less ether and more chloroform.

With apologies to the essayist, allow me to discuss local anæsthesia for operations about the nose and throat as it is carried on in the clinics of the New York Post-Graduate School and Hospital at the present time. Operations have been done without pain under cocaine anæsthesia during the past four months, in which general anæsthesia was always considered necessary. It is a daily occurrence in that institution for the antrum to be opened, the ethmoid cells curetted, the turbinates excised, septal spurs removed, and cutting operations of other sinuses done under a five or seven per cent. cocaine solution. For the complete antral operation we cocoanize as follows:

We apply the cocaine on a pledget of cotton saturated with an eight or ten per cent. solution, allowing this cotton to remain next the part to be operated upon for from fifteen to twenty minutes while we inject into the antrum a small quantity of a two or three per cent. solution. I believe all the operations that we are called upon to perform about the nose and throat can be done with a five or six per cent. solution, and we will avoid many of the disagreeable toxic effects by confining ourselves to the weaker solutions.

The application of adrenalin to the parts at the same time the cocaine is applied reduces the danger of cocaine poisoning. I apply the adrenalin and cocaine to the tissues on the same pledget of cotton, so that the combined effect is obtained. The probable action of adrenalin is the contraction of the smaller lymph- and blood-vessels, thus preventing the too rapid absorption of the cocaine.

Adrenalin is also one of the best heart tonics we have, so that it is barely possible that some of the symptoms associated with cocaine anæsthesia may be avoided by using adrenalin. There is only one feature connected with adrenalin that has proved disagreeable in my hands. There seems to be a great tendency to secondary hemorrhage, which occurs four or five hours after the operation, so that to-day I do not use it as often as when it first came on the market.

I thank you, gentlemen, for allowing me the privilege of this discussion, and the only excuse I make for introducing the subject of local anæsthesia is its common applicability to your every-day work.

*Dr. L. C. Leroy.*—How much adrenalin do you use?

*Dr. Sheedy.*—I generally apply equal parts of the one to one thousand adrenalin solution and a seven per cent. solution of cocaine to the parts on a pledget of cotton.

With regard to adrenalin, let me say that it is a drug which deteriorates very rapidly, and should be used within a short time after the bottle is opened, else it becomes acid and very irritating.

*Dr. J. Morgan Howe.*—I have nothing to say except to express my appreciation for the valuable service rendered us this evening by these gentlemen who have been so kind as to come here and tell us what we have heard, and to express my sincere thanks to them for their kindness. I would at this time move a vote of thanks to the gentlemen who have been so kind as to enlighten us upon the subect of anæsthetics.

*Dr. S. E. Davenport.*—It seems to me that considerable advance has been made in the last few years in the manner of producing general anæsthesia. My mind goes back some ten years to the occasion when a lad, especially dear to me, was anæsthetized, and so unpleasant was the experience that the boy ascribed all his subsequent illness during the slow recovery from the condition which had made an operation necessary to the bitter medicine, as he characterized it, that the doctor had forced upon him. I was informed by a lady patient the other day, that her son had recently been operated upon for suppurating mastoid processes, and that she could not say enough in commendation of the method used by the gentleman who administered the anæsthetic. The anæsthetist, evidently fond of children, made himself very agreeable, and gained the boy's confidence in a few moments. He opened his bag and invited the lad to help him take out the things. An apparatus similar to one exhibited to-night was produced, and the boy was asked if he would be willing to help "blow up this bag," and he finally went off to sleep in the midst of the game. While I am told that it is not good form to commend an essayist for what he has done for a society, I really thing that Dr. Allen, by leaving his busy life and taking the pains to bring to us such a complete exhibit of all the paraphernalia of his profession, should receive from us the very highest thanks.

*Dr. Allen.*—I should like to express my appreciation for the kind invitation and for the cordial reception and the courtesies I have been shown.

*Dr. Bogue.*—I would like to ask Dr. Sheedy how he is able to get deep local anæsthesia in the manner described, as the anæsthesia from cocaine travels from the centre towards the periphery.

*Dr. Sheedy.*—I would not attempt to give a physiological answer to the doctor's question. I only know that we do get results.

*Dr. Bogue.*—I had occasion recently to open down on to the buccal root of a molar, using the chloride of ethyl. I asked my patient if she felt it, and she did not reply. I made my operation and still she did not reply.

*Dr. Allen.*—I believe the properties of ethyl chloride were discovered while it was being used as a local anæsthetic.

*Dr. Bogue.*—I have seen it used in Paris very extensively for adenoid operations.

*Dr. Allen.*—About the dangers following etherization being underestimated, this is the point I have been trying to bring out. Both ether and chloroform have their post-operative sequellæ.

Adjourned.

FRED. L. BOGUE, M.D., D.D.S.,
*Editor The New York Institute of Stomatology.*

